# A New Prognostic Index Combines the Metabolic Response and RECIST 1.1 to Evaluate the Therapeutic Response in Patients With Non-Small Cell Lung Cancer

**DOI:** 10.3389/fonc.2020.01503

**Published:** 2020-09-02

**Authors:** Wenfang Tang, Qingyi Hou, Juntao Lin, Dongjiang Li, Jieshan Lin, Jinghua Chen, Zhenbin Qiu, Xiangpeng Chu, Xiongwen Yang, Honghong Yan, Shuxia Wang, Yilong Wu, Wenzhao Zhong

**Affiliations:** ^1^Department of Cardiothoracic Surgery, Zhongshan People's Hospital, Zhongshan, China; ^2^Guangdong Provincial Key Laboratory of Translational Medicine in Lung Cancer, Guangdong Lung Cancer Institute, Guangdong Provincial People's Hospital, Guangdong Academy of Medical Sciences, Guangzhou, China; ^3^Nuclear Medicine Department, Weilun PET Center, Guangdong Provincial People's Hospital, Guangdong Academy of Medical Sciences, Guangzhou, China; ^4^Department of Nephrology, Blood Purifiction Center, Zhongshan People's Hospital, Zhongshan, China; ^5^Guangzhou Twelfth People's Hospital, Guangzhou, China

**Keywords:** non-small cell lung cancer, positron-emission tomography, RECIST, therapeutic response, prognosis

## Abstract

**Aim:** Response Evaluation Criteria in Solid Tumors (RECIST) is occasionally insufficient for evaluation. We proposed a new prognostic index (NPI) that combines the standardized uptake value (SUV), metabolic tumor volume (MTV), and RECIST.

**Methods:** In total, 116 patients with lung cancer who underwent consecutive positron emission tomography-computed tomography prior to and after the initial treatment were included. We formulated the NPI by estimating the hazard ratios of overall survival for ΔMTV, ΔSUV_max_, and ΔD (tumor size based on RECIST). Progression-free survival (PFS) and overall survival (OS) were compared between RECIST and the NPI.

**Results:** ROC curve analysis identified two cutoff values based on the NPI (≤ −49.3% and ≥43.4%) to discriminate partial remission (NPR), stable disease (NSD) and progressive disease (NPD). Based on RECIST, survival analysis did not discriminate significantly on either PFS or OS between the PR, SD, and PD groups. However, according to the NPI, PFS and OS differed significantly between the NPR, NSD, and NPD groups (training set: PFS, *p* = 0.048; OS, *p* = 0.026; validation set: PFS, *p* = 0.004; OS, *p* = 0.023). Moreover, therapeutic response based on NPI was independent prognostic factor for both PFS [NPR as reference, NSD: hazard ratio (HR) 2.04; 95% confidence interval (95% CI) 1.35−3.08; *p* = 0.001; NPD: HR 6.87; 95% CI 3.03−15.57; *p* < 0.001] and OS (NPR as reference, NSD: HR 1.64; 95% CI 1.05−2.57; *p* = 0.031; NPD: HR 3.56; 95% CI 1.59−7.95; *p* = 0.002).

**Conclusion:** The NPI showed superiority for evaluation of the therapeutic response and survival for patients with non-small cell lung cancer, overcoming the limitations of RECIST.

## Introduction

Non-small cell lung cancer (NSCLC) is increasingly understood as a heterogeneous disease (1, 2). Treatment has been revolutionized for NSCLC patients with multimodality treatment, and their survival has been extended (3, 4). The therapeutic response in patients who receive systematic or local therapy is of interest for physicians. Currently, the standard response evaluation after treatment is based on the Response Evaluation Criteria in Solid Tumors (RECIST) (5, 6) by comparing the change in tumor size on computed tomography (CT). However, RECIST based on CT is occasionally insufficient for evaluating metabolic activity and tumor burden (7), especially with the development of targeted therapy (8–10) and immunotherapy (11–13). In patients with shrinkage at the short axis of the tumor, the RECIST were ambiguous in lesions with necrosis and fibrotic scarring after treatment; therefore, staging according to RECIST would falsely attest the disease process.

Positron emission tomography-computed tomography (PET-CT) exhibited superior advantages over CT for patients with lung cancer in staging (14, 15). It was reported that a decrease in the standardized uptake value (SUV) after treatment could predict long-term outcomes (16–19). Furthermore, metabolic tumor volume (MTV) is the volume of the tumor with an increased SUV and represents the metabolic and anatomical burden of disease, which has been found to be a predictor of recurrence (20–25). However, despite these promising findings, current clinical practice relies mainly on RECIST, and other criteria are not enough to replace it.

Consequently, we hypothesized that by combining the prognostic value of MTV and SUV_max_ with that of RECIST, we can better identify the therapeutic response of lung cancer. In this study, we propose a new prognostic index (NPI) that combines the above three variables based on the Cox proportional hazard regression model. We aimed to estimate whether the NPI can provide greater prognostic value than RECIST 1.1 alone and whether it can provide a practical and quantitative approach for clinicians to take advantage of this new prognostic index.

## Patients and Materials

### Patient Cohort

The database of non-small cell lung cancer in Guangdong Provincial People's Hospital (GDPH) was retrospectively reviewed from September 2007 to July 2015. The inclusion criteria were as follows: 1. histologically or cytologically proven lung cancer with a measurable primary tumor; and 2. PET-CT was conducted prior to and after the initiation treatment. A total of 116 patients underwent two consecutive PET-CT examinations: one for initial staging (PET_0_) and another for restaging after initial treatment (PET_1_). The time interval between PET_0_ and PET_1_ was 2.4 ± 0.8 months. The patients performed additional CT scans of the chest and other regions as clinically indicated every 3 months or with clinical suspicion of disease progression. Treatment was continued until progression was identified by CT according to RECIST 1.1, unacceptable toxicity, or patient withdrawal. The patients were randomized, with half (*n* = 58) of patients assigned to the training set and the other half (*n* =58) assigned to the validation set. The clinical T, N, and M stages were classified according to the International Association for the Study of Lung Cancer (IASLC) 8th TNM staging project (26).

### PET-CT Examinations

PET-CT scans were performed using an integrated PET-CT scanner (Gemini GXL, Philips), which was described in our previous study (27). Patients were fasted for at least 4 h, and the blood glucose level was <8.0 mmol/L before the injection of ^18^F-FDG (4.0 MBq/kg). After the administration of ^18^F-FDG, the patients rested in a comfortable environment for 1 h. Then, whole-body PET-CT images from the base of the skull to mid-thigh were acquired in a supine position with the arms raised above the head. PET, CT, and integrated PET-CT images were available to the radiologists. Interpretations of PET-CT images were made by two radiologists who were blinded to the study; a third more experienced radiologist was invited to discuss the case when different interpretations were made.

### Measurement of PET Parameters for RECIST, Positron Emission Tomography Response Criteria in Solid Tumors (PERCIST) Criteria, SUVmax, and MTV

The images from the baseline and follow-up examinations were automatically registered based on the characteristics of the bone and soft tissue on the CT scan. Using PET volume viewer software, the SUV-related parameters were automatically reviewed and compared. Two experienced PET radiologists finished separately response assessments according to the RECIST 1.1 and PERCIST 1.0 criteria, who were not blinded to the clinical information but were blinded to patient survival status and survival time.

Regarding to RECIST 1.1 criteria (5, 6), we chose up to five and no more than two lesions per organ as target lesions. We manually measured the sizes of the target lesions by the reader, and the sum percentage variation in the longest diameters of the target lesions was automatically calculated by PET volume viewer software.

As for SUVmax, we chose target lesions of RECIST 1.1 criteria with the highest ^18^F-FDG uptake for the response calculation. Furthermore, we performed measurement of MTV for target lesions of RECIST 1.1 criteria by using the volume viewer software which determined the volume of interest (VOI) using an isocontour threshold method based on SUV. MTV was defined as the tumor volume with ^18^F-FDG uptake segmented above a threshold SUV of 2.5.

For PERCIST 1.0 criteria (19), we chose the hottest lesion as the target lesion on the baseline and subsequent follow-up PET examinations. The therapeutic response assessment was based on the peak value of SUV corrected for lean body mass (SULpeak) and the liver was reference region. The regions of interest (ROIs) were manually adjusted by the reader as needed.

### Formulation of the NPI

We used a Cox proportional hazards regression model to obtain appropriate weightings for MTV [(MTV_1_−MTV_0_)/MTV_0_], SUV [(SUVmax_1_−SUVmax_0_)/SUVmax_0_] and RECIST [(D_1_−D_0_)/D_0_], respectively. D_0_, MTV_0_, and SUVmax_0_ represent the tumor size, metabolic tumor volume and maximum standardized uptake value before the initial treatment, respectively; D_1_, MTV_1_, and SUVmax_1_ represent the relative variables after the initial treatment, respectively. As a prognostic variable, the hazard ratio (HR), which was obtained from a Cox regression model of overall survival, represents an estimate of the effect on the risk (or hazard) of death from any cause. In the Cox model, all three variables were treated as continuous variables. The NPI was defined as a weighted sum of the above variables with the Cox model regression coefficients [In (HR)] as weights. The Cox regression model was fit with the method of maximum-likelihood estimation, and the estimated regression coefficients are the most likely values based on the observed data. Therefore, should provide an optimal combination of SUVmax, MTV and RECIST.

### Statistical Analysis

Statistical analyses were performed with SPSS software (version 20.0; SPSS Inc., Chicago, IL, USA). The Chi-square test or Fisher's exact test was used to compare categorical variables, and the Wilcoxon rank sum test was used for continuous variables. We drew receiver operating characteristic (ROC) curves to evaluate the diagnostic efficiency (sensitivity and specificity) for discriminating NPR, NSD, and NPD (based on the NPI, NPR: partial remission; NSD: stable disease; NPD: progressive disease), and the cutoff values of the NPI were chosen to achieve better discriminatory power in terms of NPR/NSD and NSD/NPD. Agreement of response was determined using the Kappa test. Cox proportional hazards models were used to identify prognostic factors for survival. Progression-free survival (PFS) and Overall survival (OS) curves were constructed using the Kaplan-Meier approach and compared using the log-rank test. A two-sided *p* <0.05 was considered statistically significant.

## Results

### Patient Characteristics

Across the entire patient cohort (Table 1), the median age was 62.0 years (range, 28–88 years). Overall, there were more than three times males (*n* = 93 [80.2%]) than females (*n* = 23 [19.8%]) in our study, and more than half of the patients (*n* = 66 [56.9%]) had ever smoked. Furthermore, 83 (71.6%) patients had histologically confirmed adenocarcinoma, and most of the patients (92, 79.3%) had stage IV disease. In total, 29.3% of lung cancer patients harbored an epidermal growth factor receptor (EGFR) exon 19 deletion or an exon 21 L858R mutation. Concerning the treatment strategies, 31 (26.7%) patients had initially received targeted therapy, and the other patients were treated with chemotherapy (*n* = 79 [68.1%]) or chemoradiotherapy (*n* = 6 [5.2%]). According to the RECIST 1.1, partial remission (PR), stable disease (SD), and progressive disease (PD) were confirmed in 37.9, 54.3, and 7.8% of patients, respectively. Finally, the clinical characteristics were similar between the training set and the validation set (all *p* > 0.05) (Table 1).

**Table 1:** Patient characteristics.

**Variables**	**Total, No. (%) (*n* = 116)**	**Training set, No. (%) (*n* = 58)**	**Validation set, No. (%) (*n* = 58)**	***p-*values**
Age (median, range), years	62.0 (28–88)	61.0 (28–83)	64.0 (37–88)	0.249
Gender				0.642
Male	93 (80.2)	48 (82.8)	45 (77.6)	
Female	23 (19.8)	10 (17.2)	13 (22.4)	
Smoking				0.851
Yes	66 (56.9)	32 (55.2)	34 (58.6)	
No	50 (43.1)	26 (44.8)	24 (41.4)	
Stage				0.107
IIIB	24 (20.7)	8 (13.8)	16 (27.6)	
IV	92 (79.3)	50 (86.2)	42 (72.4)	
Histology				0.839
ADC	83 (71.6)	42 (72.4)	40 (69.0)	
SCC	33 (28.4)	16 (27.6)	18 (31.0)	
Time interval between PET0 and PET1				
(Mean ± SD1), months	2.4 ± 0.8	2.3 ± 0.8	2.5 ± 0.8	0.051
EGFR types				0.969
Deletion19/ L858R	34 (29.3)	16 (27.6)	18 (31.0)	
Negative	58 (50.0)	30 (51.7)	28 (48.3)	
Unknown	24 (20.7)	12 (20.7)	12 (20.7)	
Treatment				0.806
EGFR-TKI	31 (26.7)	16 (27.6)	15 (25.9)	
CT	79 (68.1)	40 (69.0)	39 (67.2)	
CRT	6 (5.2)	2 (3.4)	4 (6.9)	
Treatment response*^a^*				0.921
PR	44 (37.9)	21 (36.2)	23 (39.7)	
SD2	63 (54.3)	32 (55.2)	31 (53.4)	
PD	9 (7.8)	5 (8.6)	4 (6.9)	

*ADC, adenocarcinoma; CRT, chemoradiotherapy; CT, chemotherapy; EGFR, epidermal growth factor receptor; MTV, metabolic tumor volume; PET, positron emission tomography; PD, progressive disease; PR, partial response; SCC, squamous cell carcinoma; SD1, standard deviation; SD2, stable disease; TKI, tyrosine kinase inhibitor*.

a *Treatment response estimated according to response evaluation criteria in solid tumors 1.1 (RECIST 1.1)*.

### Formulation of the NPI and Distribution of the NPI and RECIST [(D_1_-D_0_)/D_0_]

Estimates of the HRs and regression coefficients based on our patient cohort are shown in Table 2. We defined the NPI based on these estimates, as follows:

**Table 2:** Separated estimated hazard ratio and Cox regression coefficients for overall survival for formulation of the new prognostic index (NPI).

**Variables**	**HR (95% CI)**	**Regression coefficient**
(D_1_−D_0_)/D_0_	1.442	0.366
(MTV_1_−MTV_0_)/MTV_0_	1.051	0.050
(SUVmax_1_−SUVmax_0_)/SUVmax_0_	2.763	1.016

*D0, MTV0, SUVmax0 are the tumor size, the metabolic tumor volume and the maximum standardized uptake value before treatment, respectively; D1, MTV1, SUVmax1 are the tumor size, the metabolic tumor volume and the maximum standardized uptake value after treatment, respectively*.

*HR, Hazard Ratio; CI, confidence interval*.

NPI = 0.366^*^(D_1_−D_0_)/D_0_ + 0.050^*^(MTV_1_−MTV_0_)/MTV_0_ + 1.016^*^(SUV_max1_-SUV_max0_)/SUV_max0_.

The mean NPI value was −0.38 ± 0.56 and that of (D_1_−D_0_)/D_0_ was −0.23 ± 0.40 (Supplementary Figure S1A). Based on RECIST, the NPI in the PR, SD and PD groups was −0.80 ± 0.48, −0.21 ± 0.36 and 0.43 ± 0.59, respectively (*p* < 0.001, Supplementary Figure S1B). The change of NPI in the PR and SD groups was lower than that in the PD group.

### The Cutoff Values Among NPR, NSD and NPD Groups

Based on the NPI of the primary tumor and RECIST response, two ROC curves were deduced (Supplementary Figure S2), and cutoff values were determined to discriminate between PR and SD and between SD and PD. The area under the ROC curve (AUC) of PR and SD was 0.873 [95% confidence interval (CI) = 0.749–0.997], and the cutoff value between PR and SD was NPI ≤ −49.3%, as depicted in Supplementary Figure S2A. The sensitivity and specificity were 90.5 and 85.7%, respectively. Moreover, the AUC of SD and PD was 0.709 (95% CI = 0.396–0.990), as shown in Supplementary Figure S2B, and the cutoff value used to discriminate SD and PD was NPI ≥ 43.4%. The sensitivity and specificity were 60.0 and 90.9%, respectively. According to the cutoff values of the NPI, we defined the response of therapy by the NPI as NPR, NSD, and NPD. The patients were divided into three groups: (1) patients with an NPI ≤ −49.3% were defined as NPR; (2) those with −49.3% < NPI <43.4% were categorized as NSD; and (3) NPD included patients whose NPI was ≥43.4%.

The RECIST and NPI categories are detailed in Table 3. Patients with PR according to RECIST (44 patients) were reclassified as NPR, NSD, and NPD [38 patients (86.4%), five patients (11.4%), and one patient (2.2%), respectively]. Those with RECIST SD (63 patients) were divided into NPR [12 (19.0%)], NSD [48 (76.2%)], and NPD [3 (4.8%)] groups, respectively. Moreover, in nine patients who experienced RECIST PD, 5 (55.6%) were defined as NPD, and 4 (44.4%) were defined as NSD, respectively. According to the response based on RECIST and the NPI, the concordance rate of RECIST and the NPI was 78.4%, with a moderate agreement of treatment response (κ = 0.618, *p* < 0.001). Five patients were reclassified from PR to NSD, and 12 SD patients were reclassified to the NPR group (14.7%, 17/116), which would not influence the treatment or follow-up strategy of these patients. Furthermore, the incidence rate of discordance that led to strategy adjustment was 6.9% (8/116), which included 1 PR patients and 3 SD patients who were reclassified as NPD and transferred to the next line of therapy instead of follow-up and 4 PD patients reclassified as NSD so that their therapy would be delayed.

**Table 3:** Comparison of treatment response assessments between RECIST and NPI.

**RECIST**	**NPI**			
	**NPR**	**NSD**	**NPD**	**Total**
PR	38	5	1	44
SD	12	48	3	63
PD	0	4	5	9
Total	50	57	9	116

*NPI, new prognostic index; RECIST, response evaluation criteria in solid tumors. Based on RECIST, SD: stable disease; PR: partial response; PD: progressive disease*.

*Based on new pognostic index (NPI), NSD: stable disease; NPR: partial response; NPD: progressive disease*.

### Survival Analysis

**Figure 1:**
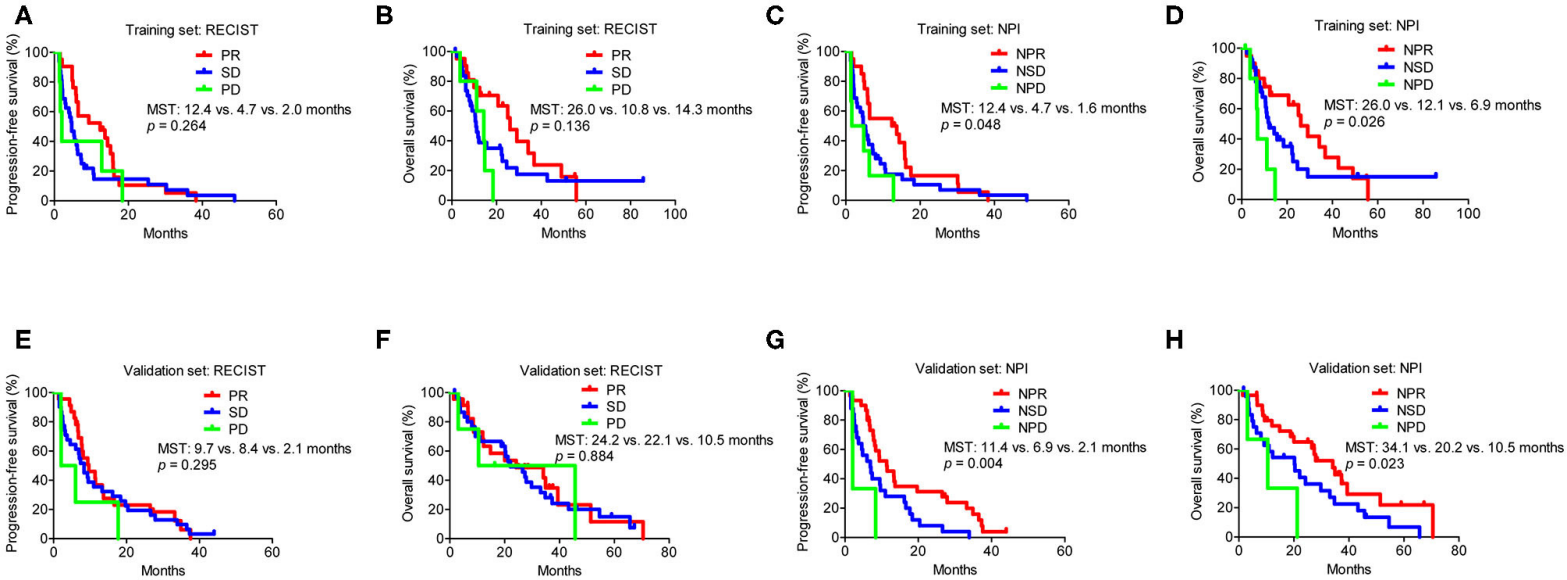
Survival analysis in subgroups according to RECIST and the NPI in the training and validation set. **(A)** Progression-free survival analysis in subgroups according to RECIST in the training set. **(B)** Overall survival analysis in subgroups according to RECIST in the training set. **(C)** Progression-free survival analysis in subgroups according to NPI in the training set. **(D)** Overall survival analysis in subgroups according to NPI in the training set. **(E)** Progression-free survival analysis in subgroups according to RECIST in the validation set. **(F)** Overall survival analysis in subgroups according to RECIST in the validation set. **(G)** Progression-free survival analysis in subgroups according to NPI in the validation set. **(H)** Overall survival analysis in subgroups according to NPI in the validation set. MST, median survival time; NPI, new prognostic index; RECIST, Response Evaluation Criteria in Solid Tumors. Based on RECIST, PR: partial response; SD: stable disease; and PD: progressive disease. Based on the NPI, NPR: partial response; NSD: stable disease; and NPD: progressive disease.

The median follow-up time was 14.5 (range, 1.4–85.7) months, and the last follow-up was recorded on April 10, 2017. The clinical characteristics between the three subgroups (PR, SD, and PD) based on RECIST or the three subgroups (NPR, NSD, and NPD) according to the NPI were shown in Supplementary Table S1. Based on RECIST, the patients in PD group were younger than the patients in PR and SD groups (*p* = 0.046). Moreover, lung squamous cell carcinoma was more common in PR (*p* = 0.001) and NPR groups (*p* = 0.009). All other clinical characteristics were similar between the PR, SD, and PD groups and between the NPR, NSD, and NPD groups, respectively. In the training set, based on RECIST 1.1, survival analysis revealed no significant difference in either PFS or OS between the PR, SD and PD groups (PR vs. SD vs. PD groups: PFS, 12.4 vs. 4.7 vs. 2.0 months, *p* = 0.264; OS, 26.0 vs. 10.8 vs. 14.3 months, *p* = 0.136) (Figures 1A,B). And the results were similar for PFS (*p* = 0.295) and OS (*p* = 0.884) in the validation set (Figures 1E,F). For PERCIST criteria, both in training and validation set, the PFS were significantly different between PMR, SMD and PMD groups (training set: 12.4 vs. 3.9 vs. 1.4 months, *p* = 0.020; validation set: 11.4 vs. 4.8 vs. 2.1, *p* = 0.004). For OS based on PERCIST criteria, the results showed significant difference in training set (26.0 vs. 10.5 vs. 6.9 months, *p* = 0.008), but it did not distinguish significantly survival benefit in validation set (27.4 vs. 12.3 vs. 10.5 months, *p* = 0.302). Importantly, there was no statistical difference for survival between SMD and PMD group in any population subset (Supplementary Figure S3).

However, according to the NPI, the PFS differed significantly between the NPR, NSD, and NPD groups in the training set (12.4 vs. 4.7 vs. 1.6 months, *p* = 0.048), which was similar to that observed in the validation set (11.4 vs. 6.9 vs. 2.1 months, *p* = 0.004) (Figures 1C,G). Moreover, there was also a significant difference for OS based on the NPI in both sets (NPR vs. NSD vs. NPD groups: training set, 26.0 vs. 12.1 vs. 6.9 months, *p* = 0.026; validation set, 34.1 vs. 20.2 vs. 10.5 months, *p* = 0.023) (Figures 1D,H).

Furthermore, when stratifying by different treatments, compared with RECIST, the NPI also showed superiority to assess survival benefit according to different therapeutic responses in targeted therapy and chemotherapy cohorts. Whereas, due to small number of patients in chemoradiotherapy group (*n* = 6), there was no significant difference to distinguish survival benefit based on RECIST and NPI (Supplementary Figure S4).

By using Cox proportional hazards models, univariate and multivariable analyses were then performed to investigate prognostic factors associated with PFS and OS. Based on NPI, we found that EGFR types (*p* < 0.001) and therapeutic response based on NPI (*p* < 0.001) were independent prognostic factor for PFS (Table 4). Furthermore, female sex (*p* = 0.001) and therapeutic response based on NPI (*p* = 0.004) were independent prognostic factors for OS (Table 4). These results indicated that the NPI could predict survival independently, however, the therapeutic response based on RECIST was not independent prognostic factor for both PFS and OS (Table 5).

**Table 4:** Cox regression based on NPI in total cohort.

**Variables**	**PFS**				**OS**			
	**Univariate analysis**		**Multivariable analysis**		**Univariate analysis**		**Multivariable analysis**	
	**HR (95% CI)**	***p-*values**	**HR (95% CI)**	***p-*values**	**HR (95% CI)**	***p-*values**	**HR (95% CI)**	***p-*values**
Female sex	0.50 (0.31–0.81)	**0.005**			0.33 (0.18–0.61)	**<0.001**	0.35 (0.19–0.66)	**0.001**
>65 years age	0.66 (0.44–0.98)	**0.042**			0.83 (0.53–1.30)	0.417		
Smoking history	1.30 (0.88–1.90)	0.186			2.01 (1.29–3.16)	**0.002**		
Stage IV	1.21 (0.76–1.94)	0.425			1.30 (0.77–2.19)	0.322		
SCC	0.93 (0.61–1.42)	0.732			0.95 (0.59–1.51)	0.821		
EGFR types		**0.007**		**<0.001**		**0.034**		
Wild-type	1		1		1			
Mutation	0.50 (0.32–0.78)	**0.002**	0.37 (0.23–0.59)	**<0.001**	0.53 (0.32–0.88)	**0.013**		
Unknown	0.65 (0.40–1.07)	0.089	0.69 (0.42–1.13)	0.143	0.65 (0.37–1.15)	0.143		
Treatments		**0.010**				0.128		
EGFR-TKI	1				1			
CT	1.71 (1.11–2.64)	**0.015**			1.47 (0.90–2.40)	0.124		
CT+RT	0.56 (0.20–1.59)	0.273			0.61 (0.18–2.04)	0.420		
Therapeutic response based on NPI		**<0.001**		**<0.001**		**0.001**		**0.004**
NPR	1		1		1		1	
NSD	1.79 (1.20–2.66)	**0.004**	2.04 (1.35–3.08)	**0.001**	1.67 (1.07–2.62)	**0.025**	1.64 (1.05–2.57)	**0.031**
NPD	3.75 (1.80–7.83)	**<0.001**	6.87 (3.03–15.57)	**<0.001**	4.32 (1.94–9.63)	**<0.001**	3.56 (1.59–7.95)	**0.002**

*ADC, adenocarcinoma; CI, confidence interval; CT, chemotherapy; EGFR, epidermal growth factor receptor; HR, hazard ratio; NPI, new prognostic index; OS, overall survival; PFS, progression-free survival; RT, radiotherapy; SCC, squamous cell carcinoma; TKI, tyrosine kinase inhibitor*.

*Based on the NPI, NPR, partial response; NSD, stable disease; NPD, progressive disease*.

*The bold values meant p < 0.05*.

**Table 5:** Cox regression based on RECIST in total cohort.

**Variables**	**PFS**				**OS**			
	**Univariate analysis**		**Multivariable analysis**		**Univariate analysis**		**Multivariable analysis**	
	**HR (95% CI)**	***p-*values**	**HR (95% CI)**	***p-*values**	**HR (95% CI)**	***p-*values**	**HR (95% CI)**	***p-*values**
Female sex	0.50 (0.31–0.81)	**0.005**	0.46 (0.27–0.77)	**0.004**	0.33 (0.18–0.61)	**<0.001**	0.40 (0.19–0.85)	**0.018**
> 65y age	0.66 (0.44–0.98)	**0.042**	0.61 (0.40–0.92)	**0.019**	0.83 (0.53–1.30)	0.417		
Smoking history	1.30 (0.88–1.90)	0.186			2.01 (1.29–3.16)	**0.002**		
Stage IV	1.21 (0.76–1.94)	0.425			1.30 (0.77–2.19)	0.322		
SCC	0.93 (0.61–1.42)	0.732			0.95 (0.59–1.51)	0.821		
EGFR types		**0.007**				**0.034**		
Wild–type	1				1			
Mutation	0.50 (0.32–0.78)	**0.002**			0.53 (0.32–0.88)	**0.013**		
Unknown	0.65 (0.40–1.07)	0.089			0.65 (0.37–1.15)	0.143		
Treatments		**0.010**		**0.028**		0.128		
EGFR–TKI	1		1		1			
CT	1.71 (1.11–2.64)	**0.015**	1.48 (0.94–2.33)	0.091	1.47 (0.90–2.40)	0.124		
CT+RT	0.56 (0.20–1.59)	0.273	0.46 (0.15–1.38)	0.165	0.61 (0.18–2.04)	0.420		
Therapeutic response based on RECIST		0.163				0.233		
PR	1				1			
SD	1.29 (0.87–1.93)	0.209			1.27 (0.81–2.00)	0.302		
PD	1.92 (0.93–3.97)	0.079			1.93 (0.88–4.25)	0.101		

*ADC, adenocarcinoma; CI, confidence interval; CT, chemotherapy; EGFR, epidermal growth factor receptor; HR, hazard ratio; RECIST, response evaluation criteria in solid tumors; OS, overall survival; PFS, progression-free survival; RT, radiotherapy; SCC, squamous cell carcinoma; TKI, tyrosine kinase inhibitor*.

*Based on the RECIST, PR, partial response; SD, stable disease; PD, progressive disease*.

*The bold values meant p <0.05*.

## Discussion

In this study, we evaluated the efficacy of an NPI in predicting the therapeutic response of non-small cell lung cancer. We found that the response based on the NPI was prognostic factor for PFS and OS. Compared to RECIST 1.1, the results revealed that the NPI overcame the limitation of RECIST in estimating the activity of tumor.

For the treatment of lung cancer, early response evaluation provides guidance for appropriate individual therapy. In addition, survival depends on the biological behavior of the tumor rather than the residual tumor size after treatment (7, 28). A residual tumor after treatment may contain inflammatory tissue, fibrotic tissue or resistant clones, and CT evaluation based on size is insufficient for these diseases. Currently, two protocols regarding the therapeutic response in terms of metabolic changes have been described: in 1999, the European Organization for Research and Treatment of Cancer (EORTC) (29) recommended the change of SUVbsa (SUV normalized to body surface area), and in 2009, Wahl et al. (19) recommended that SUL (SUV corrected to lean body mass) be the indicator of response (PERCIST criteria). Investigators (16) have concentrated on comparing the efficacy of the RECIST, EORTC and PERCIST criteria, and the results have indicated that the metabolic response based on the PERCIST was more sensitive and accurate in predicting response and was at least as effective as RECIST in predicting survival. Additionally, MTV based on PET-CT further accurately assessed the metabolic activity and morphologic parameters of the tumor. Chung et al. (23) suggested a significant prolongation of PFS (17.0 vs. 4.5 months, *p* < 0.001) and OS (44.0 vs. 14.0 months, *p* < 0.001) in advanced lung adenocarcinoma with a low MTV (< 90). In a prospective study launched by Huang et al. (30), local recurrence-free survival was significantly longer in patients with a decreased MTV over 29.7% than those with a decreased MTV <29.7% after concurrent chemoradiotherapy for locally advanced NSCLC (35 vs. 13 months, *p* <0.001). However, the above two variables do not propose a rigorous standardization protocol or involve the performance of cumbersome tasks.

Therefore, we proposed a new prognostic index (NPI) combining the metabolic response (MTV and SUV_max_) and anatomical changes [RECIST: (D_1_−D_0_)/D_0_)] to better differentiate therapeutic responses. In our study, survival was significantly longer in the NPR group than in the NSD or NPD group. Compared with groups defined by RECIST, the discriminatory ability based on the NPI was better, either in training and validation set or targeted therapy and chemotherapy cohorts. These results revealed the competitive ability of the NPI to extract patients with favorable survival from those with poor survival. The NPI may be more accurate in the adjustment of therapy to avoid unnecessary or delayed therapy and may lighten the economic or mental burden of the patients.

The current study had some limitations. This was a retrospective study within a single academic institution and a small population. Retrospective validation with data from other institutions and prospective validation are needed to further establish the prognostic value of the NPI. Moreover, further study with larger population should be focus on different treatment strategies, especially for immunotherapy. However, we emphasized the combination of tumor biology and anatomical changes in evaluating therapeutic response of non-small cell lung cancer and proposed a new approach for clinical practice.

In conclusion, the NPI that we propose is a combination of the MTV, SUV_max_, and RECIST. It provides a novel approach for clinicians to combine the prognostic values of metabolic response and anatomical changes for non-small cell lung cancer and achieves improved prognostic and therapy accuracy compared with current practice based on RECIST.

## Data Availability Statement

The raw data supporting the conclusions of this article will be made available by the authors, without undue reservation.

## Ethics Statement

The studies involving human participants were reviewed and approved by This study was approved by the Ethics and Scientific Committees of Guangdong Provincial People's Hospital [No. GDREC2016175H(R2)]. The patients/participants provided their written informed consent to participate in this study.

## Author Contributions

WT, QH, JuL, DL, and WZ contributed to conceptualization, study design, manuscript preparation, and editing. JC, JiL, XC, and ZQ contributed to collect the data of patients and followed up. WT, XY, and HY contributed to data analysis. SW, YW, and WZ contributed to the interpretation of the statistical results and manuscript revising. All authors approved the final submitted manuscript.

## Conflict of Interest

The authors declare that the research was conducted in the absence of any commercial or financial relationships that could be construed as a potential conflict of interest.
