# Supplementary material for: A New Prognostic Index Combines the Metabolic Response and RECIST 1.1 to Evaluate the Therapeutic Response in Patients With Non-Small Cell Lung Cancer
Source: Front Oncol. 2020 Sep 2;10:1503. doi: 10.3389/fonc.2020.01503 (PMC7493745; doi:10.3389/fonc.2020.01503)
Supplement: Supplementary file 1 [file Data_Sheet_1.pdf]

Supplementary materials

Table S1. Patient characteristics between different subgroups.

Figure S1. (A) Percentage change according to RECIST and the NPI; (B) The NPI in different RECIST groups. NPI, new prognostic index; RECIST, Response Evaluation Criteria in Solid Tumors; Based on RECIST, PR: partial response; SD: stable disease; and PD: progressive disease.

Figure S2. ROC curves of the NPI for RECIST (Figure S2A: PR and SD; S2B: SD and PD). NPI, new prognostic index; Based on the RECIST, PR: partial response; SD: stable disease; and PD: progressive disease; RECIST, Response Evaluation Criteria in Solid Tumors; ROC, receiver operating characteristic.

Figure S3. Survival analysis in subgroups according to PERCIST criteria in the training and validation set. MST, median survival time; PERCIST: Positron Emission Tomography Response Criteria in Solid Tumors. Based on PERCIST, PMR: partial response; SMD: stable disease; and PMD: progressive disease

Figure S4. Survival analysis based on RECIST and NPI stratified by EGFR-TKI, chemotherapy and chemoradiotherapy cohorts. EGFR, epidermal growth factor receptor; MST, median survival time; NPI, new prognostic index; RECIST, Response Evaluation Criteria in Solid Tumors; TKI, tyrosine kinase inhibitor. Based on RECIST, PR: partial response; SD: stable disease; and PD: progressive disease. Based on the NPI, NPR: partial response; NSD: stable disease; and NPD: progressive disease.

Table S1. Patient characteristics between different subgroups.

	RECIST				NPI			
Variables	PR, No.(%) (n = 44)	SD, No.(%) (n = 63)	PD, No.(%) (n = 9)	*p* values	NPR, No.(%) (n = 50)	NSD, No.(%) (n = 57)	NPD, No.(%) (n = 9)	*p* values
Age (Median, range), years	63.0 (32-81)	62.0 (28-88)	53.0 (34-64)	**0.046**	63.0 (39-88)	61.0 (28-87)	53.0 (34-78)	0.162
Gender				0.487				0.333
Male	37 (84.1)	48 (76.2)	8 (88.9)		39 (78.0)	45 (78.9)	9 (100.0)	
Female	7 (15.9)	15 (23.8)	1 (11.1)		11 (22.0)	12 (21.1)	0 (0.0)	
Smoking				0.744				0.549
Yes	26 (59.1)	36 (57.1)	4 (44.4)		27 (54.0)	35 (61.4)	4 (44.4)	
No	18 (40.9)	27 (42.9)	5 (55.6)		23 (46.0)	22 (38.6)	5 (55.6)	
Stage				0.333				0.685
IIIB	11 (25.0)	10 (15.9)	3 (33.3)		12 (24.0)	11 (19.3)	1 (11.1)	
IV	33 (75.0)	53 (84.1)	6 (66.7)		38 (76.0)	46 (80.7)	8 (88.9)	
Histology				**0.001**				**0.009**
ADC	22 (50.0)	53 (84.1)	7 (77.8)		28 (56.0)	47 (82.5)	7 (77.8)	
SCC	22 (50.0)	10 (15.9)	2 (22.2)		22 (44.0)	10 (17.5)	2 (22.2)	
Time interval between PET_0 and PET_1 (Mean ± SD), months	2.5 ± 0.8	2.3 ± 0.8	2.7 ± 0.9	0.182	2.5 ± 0.8	2.3 ± 0.8	2.6 ± 1.1	0.317
EGFR types				0.270				0.223
Deletion19/ L858R	11 (25.0)	20 (31.7)	3 (33.3)		15 (30.0)	14 (24.6)	5 (55.6)	
Negative	19 (43.2)	34 (54.0)	5 (55.6)		22 (44.0)	32 (56.1)	4 (44.4)	
Unknown	14 (31.8)	9 (14.3)	1 (11.1)		13 (26.0)	11 (19.3)	0 (0.0)	
Treatment				0.472				0.100
EGFR-TKI	11 (25.0)	16 (25.4)	4 (44.4)		13 (26.0)	13 (22.8)	5 (55.6)	
CT	29 (66.0)	45 (71.4)	5 (55.6)		32 (64.0)	43 (75.4)	4 (44.4)	
CRT	4 (9.0)	2 (3.2)	0 (0.0)		5 (10.0)	1 (1.8)	0 (0.0)	

ADC; adenocarcinoma; CRT, chemoradiotherapy; CT: chemotherapy; EGFR, epidermal growth factor receptor; MTV, metabolic tumor volume; NPI, new prognostic index; NPR: partial response based on NPI; NSD: stable disease based on NPI; NPD: progressive disease based on NPI; PD, progressive disease; PET, positron emission tomography; PR, partial response; RECIST, response evaluation criteria in solid tumors; SCC, squamous cell carcinoma; SD, standard deviation; TKI, tyrosine kinase inhibitor.

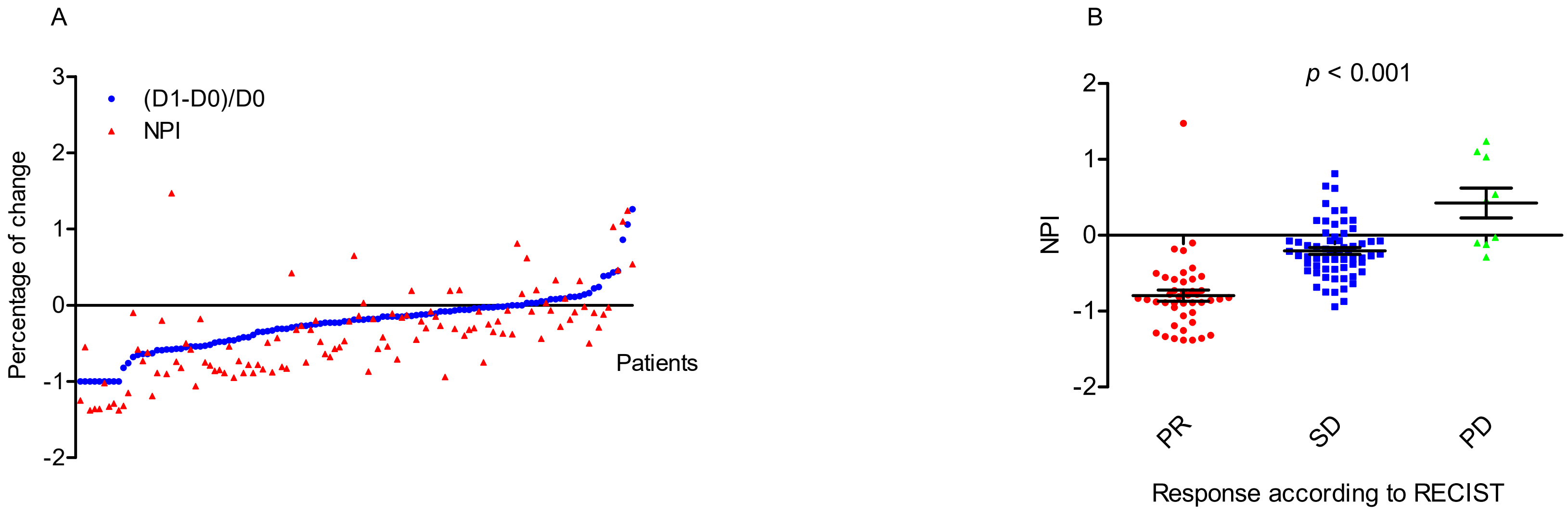
A
(D1-D0)/D0
NPI
Percentage of change
3
2
1
0
-1
-2
Patients
B
p < 0.001
NPI
2
1
0
-1
-2
PR
SD
PD
Response according to RECIST

Figure S1

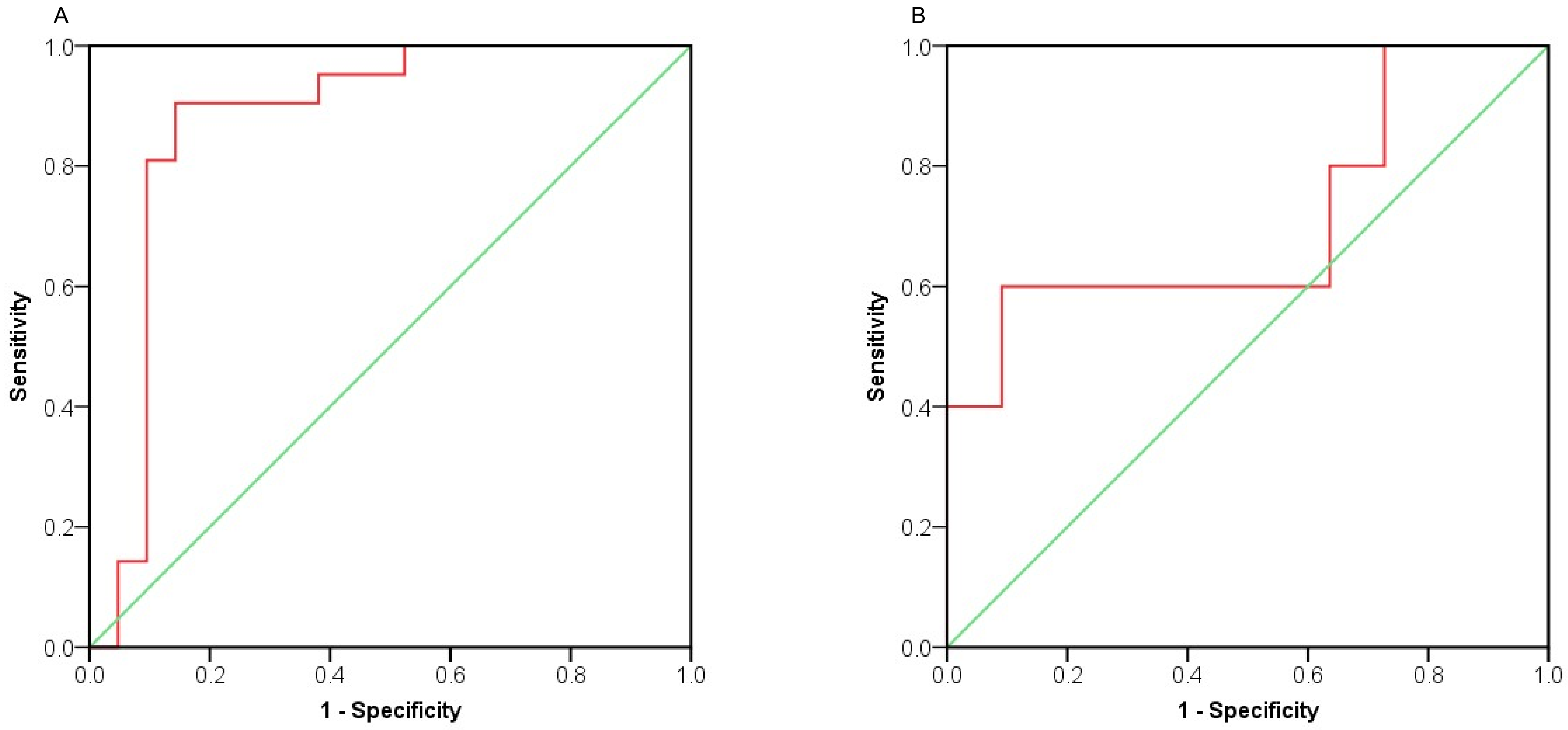

Figure S2

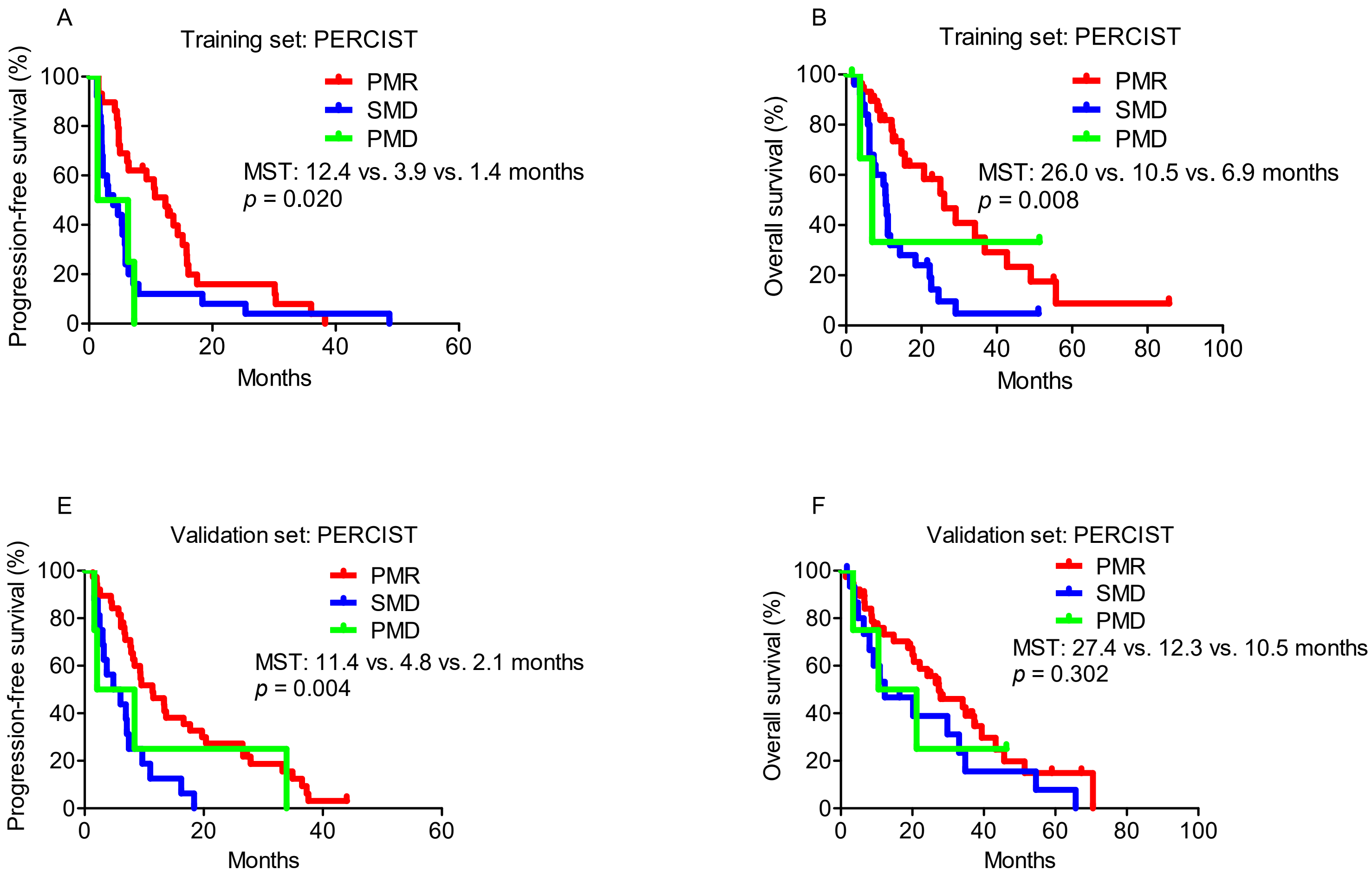
A
Training set: PERCIST
Progression-free survival (%)
PMR
SMD
PMD
MST: 12.4 vs. 3.9 vs. 1.4 months
p = 0.020
Months
B
Training set: PERCIST
Overall survival (%)
PMR
SMD
PMD
MST: 26.0 vs. 10.5 vs. 6.9 months
p = 0.008
Months
E
Validation set: PERCIST
Progression-free survival (%)
PMR
SMD
PMD
MST: 11.4 vs. 4.8 vs. 2.1 months
p = 0.004
Months
F
Validation set: PERCIST
Overall survival (%)
PMR
SMD
PMD
MST: 27.4 vs. 12.3 vs. 10.5 months
p = 0.302
Months

Figure S3

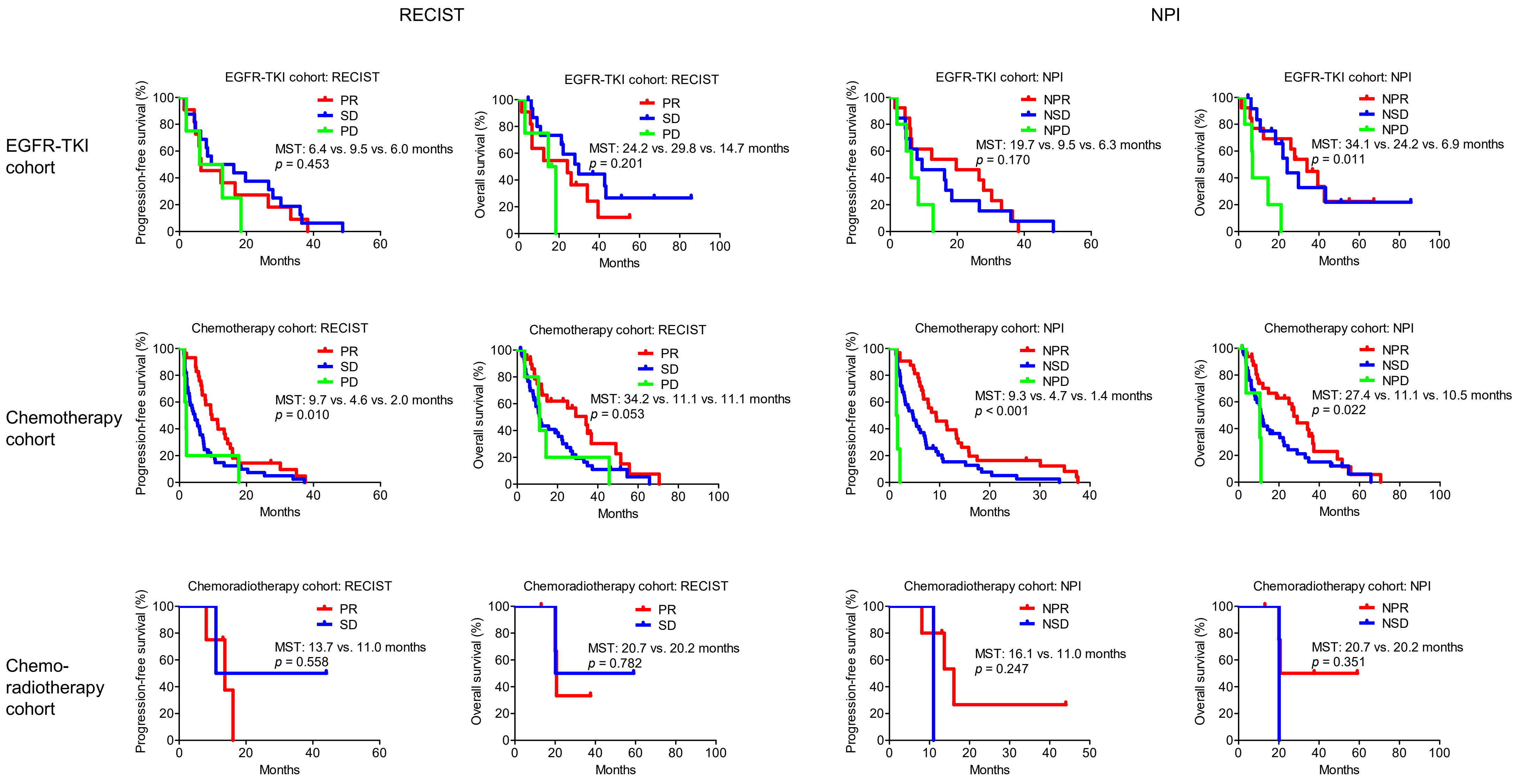
RECIST
NPI
EGFR-TKI cohort
Chemotherapy cohort
Chemo-radiotherapy cohort
EGFR-TKI cohort: RECIST
Progression-free survival (%)
Months
PR
SD
PD
MST: 6.4 vs. 9.5 vs. 6.0 months
p = 0.453
EGFR-TKI cohort: RECIST
Overall survival (%)
MST: 24.2 vs. 29.8 vs. 14.7 months
p = 0.201
EGFR-TKI cohort: NPI
NPR
NSD
NPD
MST: 19.7 vs. 9.5 vs. 6.3 months
p = 0.170
EGFR-TKI cohort: NPI
MST: 34.1 vs. 24.2 vs. 6.9 months
p = 0.011
Chemotherapy cohort: RECIST
MST: 9.7 vs. 4.6 vs. 2.0 months
p = 0.010
Chemotherapy cohort: RECIST
MST: 34.2 vs. 11.1 vs. 11.1 months
p = 0.053
Chemotherapy cohort: NPI
MST: 9.3 vs. 4.7 vs. 1.4 months
p < 0.001
Chemotherapy cohort: NPI
MST: 27.4 vs. 11.1 vs. 10.5 months
p = 0.022
Chemoradiotherapy cohort: RECIST
MST: 13.7 vs. 11.0 months
p = 0.558
Chemoradiotherapy cohort: RECIST
MST: 20.7 vs. 20.2 months
p = 0.782
Chemoradiotherapy cohort: NPI
MST: 16.1 vs. 11.0 months
p = 0.247
Chemoradiotherapy cohort: NPI
MST: 20.7 vs. 20.2 months
p = 0.351

Figure S4
